# The mitochondrial genome of *Anthalia* sp. (Diptera: Empididae)

**DOI:** 10.1080/23802359.2020.1775523

**Published:** 2020-06-12

**Authors:** Chenjing Zhao, Runhua Li, Shang Gao, Ding Yang

**Affiliations:** a Department of Biology, Taiyuan Normal University, Jinzhong, China; b College of Plant Protection, China Agricultural University, Beijing, China

**Keywords:** Mitochondrial genome, Ocydromiinae, phylogenetics

## Abstract

The dance fly *Anthalia* sp. belongs to the subfamily Ocydromiinae of Empididae. The mitogenome (GenBank accession number: MT483943) of *Anthalia* sp. was sequenced, the new representative of the mitogenome of the subfamily. The nearly complete mitogenome is 15,142 bp totally, consisting of 13 protein-coding genes, 2 rRNAs, and 22 transfer RNAs. All genes have the similar locations and strands with that of other published species of Empididae. The nucleotide composition biases toward A and T, which together made up 78.6% of the entirety. Bayesian inference analysis strongly supported the monophyly of Empidoidea, Empididae, and Dolichopodidae. It is clear that the phylogenetic relationship within Empidoidea: (Dolichopodinae + Neurigoninae) + ((Empidinae + (Trichopezinae + Oreogetoninae)) + Ocydromiinae) in this study.

## Introduction

Family Empididae is distributed worldwide with over 5000 known species. It is a useful natural enemy insect resource as their adults and larvae are predatory upon crop and health pests (Yang et al. 2007).

The specimens of *Anthalia* sp. used for this study were collected in Gongshan County of Yunnan by Bing Zhang and identified by Ding Yang. Specimens are deposited in the Entomological Museum of China Agricultural University (CAU) with the accession number is CAUYD3030 (Plant Protection Building, West Campus, China Agricultural University). The total genomic DNA was extracted from the whole body (except head) of the specimen using the QIAamp DNA Blood Mini Kit (Qiagen, Germany) and stored at −20 °C until needed. The mitogenome was sequenced in BaiNuoDaCheng biotechnology company used NGS. The nearly complete mitogenome of *Anthalia* sp. is 15,142 bp (GenBank accession number: MT483943). It encoded 13 PCGs, 22 tRNA genes, and 2 rRNA genes and were similar with related reports before (Hou et al. 2019; Qilemoge et al. 2019; Yang et al. 2019; Qilemoge et al. 2020). All genes have similar locations and strands with that of other published Empididae species. The nucleotide composition of the mitogenome was biased toward A and T, with 78.6% of A + T content (A = 39.8%, T = 38.8%, C = 12.3%, G = 9.1%). The A + T content of PCGs, tRNAs, and rRNAs is 77.3, 79.1, and 82.4% respectively. The total length of all 13 PCGs of *Anthalia* sp. is 11,214 bp. Five PCGs (*NAD2*, *ATP8, NAD3, NAD5, NAD6*) initiated with ATT codons, and five PCGs (*COII*, *COIII*, *ATP6*, *NAD4*, and *CYTB*) initiated with ATG codons, and *COI* initiated with TCG as a start codon, and *NAD4L* and *NAD1* initiated with ATA as a start codon. All 13 PCGs used the typical termination codons TAA in *Anthalia* sp.

**Figure 1.**
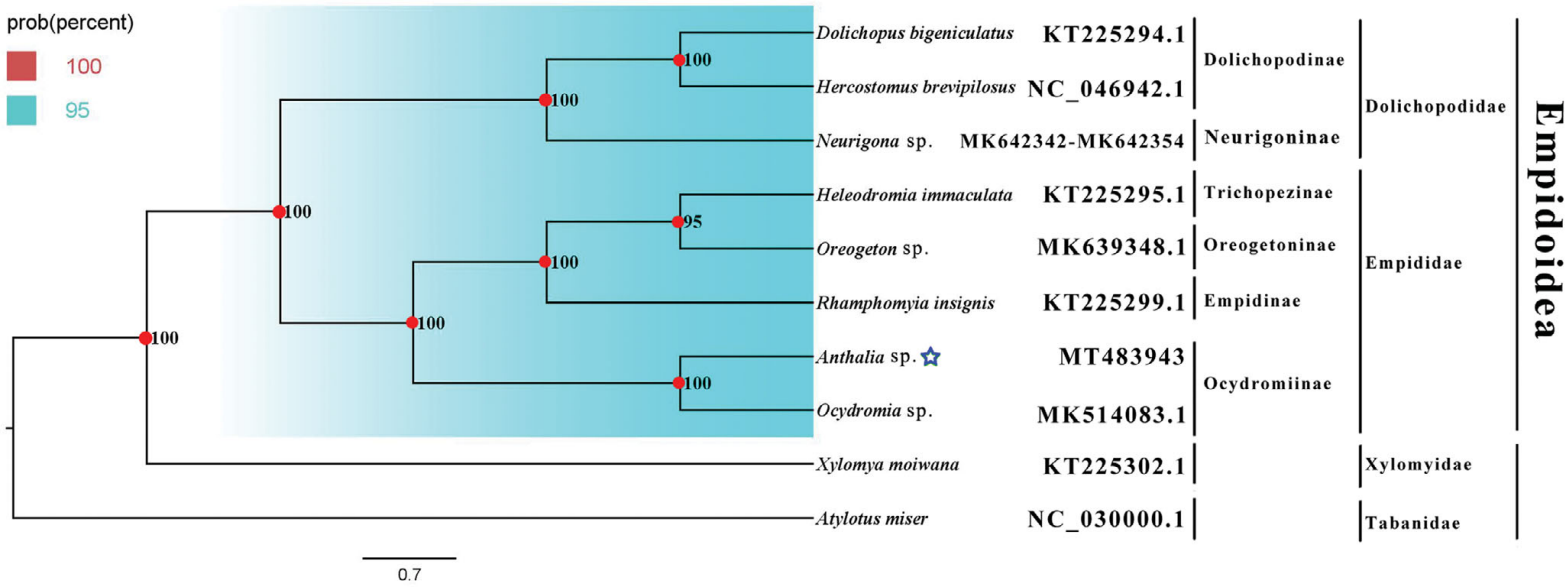
Bayesian phylogenetic tree of 10 Diptera species. The posterior probabilities are labeled at each node. Genbank accession numbers of all sequence used in the phylogenetic tree have been included in Figure 1 and corresponding to the names of all species.

Phylogenetic analysis was performed based on the nucleotide sequences of 13 PCGs from 10 Diptera species. Bayesian (BI) analysis generated the phylogenetic tree topologies based on the PCGs matrices (Figure 1). The phylogenetic result shows that the monophyly of Empidoidea, Dolichopodidae, and Empididae were strongly supported. The monophyletic Dolichopodidae that contains Dolichopodinae and Neurigoninae was assigned to the sister group to the clade of Empididae that consists of Empidinae, Trichopezinae, Oreogetoninae, and Ocydromiinae in this study. It is clear that the phylogenetic relationship within Empidoidea: (Dolichopodinae + Neurigoninae) + ((Empidinae + (Trichopezinae + Oreogetoninae)) + Ocydromiinae). This result shows that Empididae and Dolichopodidae are monophyletic, respectively, which is consistent with the phylogenetic result of the previous research (Wang et al. 2016). The mitogenome of *Anthalia* sp. could provide important information for the further studies of Empidoidea phylogeny.

## Data Availability

The data that support the findings of this study are openly available in [NCBI] at [https://www.ncbi.nlm.nih.gov/], reference number [MT483943].
